# Grass Carp (*Ctenopharyngodon idellus*) NIMA-Related Kinase 6 Blocks dsRNA-Induced IFN I Response by Targeting IRF3

**DOI:** 10.3389/fimmu.2020.597775

**Published:** 2021-01-08

**Authors:** Xiaowen Xu, Meifeng Li, Zeyuan Deng, Jihuan Hu, Zeyin Jiang, Yapeng Liu, Kaile Chang, Chengyu Hu

**Affiliations:** ^1^ College of Life Science, Nanchang University, Nanchang, China; ^2^ State Key Laboratory of Food Science and Technology, Nanchang University, Nanchang, China

**Keywords:** NEK6, IRF3-mediated antiviral responses, IFN I, inhibitor, fish

## Abstract

Accumulating evidence indicates that mammalian NIMA (never in mitosis, gene A)-related kinase 6 (NEK6) plays potential roles during the course of tumorigenesis, but little is known about NEK6 in lower vertebrates. Herein, we reported a mammalian ortholog of NEK6 in grass carp (*Ctenopharyngodon idellus*) (CiNEK6). Multiple alignment of amino acid sequences and phylogenetic analysis showed that CiNEK6 shares a high level of sequence similarity with its counterparts in birds. CiNEK6 was ubiquitously expressed in all tested tissues, and its expression level was increased under treatment with GCRV (dsRNA virus) or poly I:C (dsRNA analog). Q-PCR and dual-luciferase assays suggested that CiNEK6 overexpression suppressed IFN I activity in CIK cells treated with poly I:C. Knockdown of CiNEK6 resulted in a higher level of IFN I expression in CIK cells treated with poly I:C compared to those which received PBS. Interestingly, analysis of subcellular localization demonstrated that CiNEK6 protein scattered throughout the cytoplasm is gradually congregated together at the edges of karyotheca upon stimulation with poly I:C. Co-IP and co-localization assays suggested that CiNEK6 interacts with CiIRF3 after poly I:C challenge. In poly I:C-treated cells, the phosphorylation of CiIRF3 was increased by CiNEK6 knockdown, but was suppressed by CiNEK6 overexpression, suggesting that CiNEK6 decreases IFN I expression through inhibiting CiIRF3 activity. Cell viability assay, crystal violet staining, and detection of Vp5 also showed that CiNEK6 plays an inhibitory role in IRF3-mediated antiviral responses.

## Introduction

The innate immune responses are well developed in vertebrates to fight off various viral infections during their long battle with foreign pathogens (1, 2). In host cells, PRRs (pattern recognition receptors) are expressed to specifically recognize PAMPs (pathogen-associated molecular patterns), and then activate various innate immune or inflammatory responses (3, 4). To date, a variety of PRRs have been identified, including TLRs (Toll-like receptors), RLRs (RIG-I like receptors), NLRs (NOD-like receptors) and CDRs (cytoplasmic DNA receptors) (5–8).

Although the innate immune system plays a critical role in the host defense against virus infection, overactive immune response results in autoimmune disease. Actually, several negative regulators have been identified in TLRs and RLRs-mediated pathways, for instance, RP105 blocks the formation of TLR4-LPS complex and inhibits inflammatory response (9); ST2L captures MyD88 and TIRAP, which suppresses TLR4-mediated pathway (10); RNF5 negatively regulates virus-triggered IRF3 activation and antiviral response *via* targeting MITA for ubiquitination and degradation (11); DAK inhibits MDA5-mediated pathway through specifically binding to CARD domain of MDA5 (12).

NIMA-related kinase family consists of 11 members (from NEK1 to NEK11), which mainly participate in the regulation of mitosis (13). NEK6 is identified in mitotic cells with a high level of similarity with NEK7 (14). NEK6 is up-regulated in malignant tumors and cancer cells; therefore, it is regarded as a pivotal regulatory factor in tumorigenesis (15, 16). Recent studies indicated that NEK6 suppresses the nuclear translocation of Smad4 and then inhibits the activity of TGFβ (17), indicating that NEK6 acts as an inhibitory regulator of the TGFβ-Smad signaling pathway. Moreover, the down-regulation of NEK6 initiates the p53-induced premature senescence (18). These previous studies showed that the function of NEK6 is complicated in several cellular pathways. Therefore, it is necessary to determine the detailed roles which NEK6 plays in cellular signaling pathway.

Just like in mammals, there is also positive innate immune response in fish to fight against pathogenic infection. However, the negative regulatory mechanism is rarely reported in fish. Grass carp IRF2 serves as a negative regulator for IRF1-induced IFN response (19). Grass carp LGP2 antagonizes RIG-I or MDA5-mediated antiviral response in resting state and early stage of GCRV infection (20). Zebrafish TBK1 isoforms inhibit the formation of TBK1-IRF3 complex and IRF3 phosphorylation (21). More work is hence needed to investigate the negative regulatory mechanism of innate immunity in fish.

In the present study, a NEK6 orthologous gene (MT 668702) is identified in grass carp (CiNEK6). It is found that CiNEK6 suppresses IFN I expression and antiviral activity through blocking IRF3 phosphorylation in response to the stimulation with dsRNA (poly I:C or GCRV), suggesting that CiNEK6 serves as a negative regulator for IRF3-induced IFN I response.

## Materials and Methods

### Fish, Cell Lines, and Virus

Grass carp (mean weight, 20 g) were kindly donated from Ganzhou Fisheries Institute (Ganzhou, China). The fish were raised in aerated freshwater for 14 days prior to experiments. *C. idellus* kidney (CIK) cells and *C. idellus* ovary (CO) cells were stable cell lines and cultivated in M199 medium containing 10% FBS at 28°C. Poly I:C, a kind of dsRNA analog, was purchased from Sigma (USA) and diluted in M199 medium.

GCRV is a member of genus Aquareovirus (dsRNA virus) in the family Reoviridae, which is the first viral pathogen to be identified from aquatic animals in China in 1983 (22). GCRV 097, a strain of GCRV virus, was propagated in CIK cells. In detail, CIK cells were seeded in 10-cm dishes for 12 h, and then infected with 100 μl 10^−8^ TCID_50_ GCRV. After 2 h, virus inoculum was removed and the cells were cultivated with new M199 medium. Five days later, the virus was harvested through multigelation and virus titer was analyzed according to the method of Reed and Muench.

### Cloning of a Full-Length cDNA Encoding C. idellus NEK6

The homologous fragment of *CiNEK6* was obtained from GCGD server (http://bioinfo.ihb.ac.cn/gcgd/php/index.php). RACE-PCR was used to amplify the whole cDNA sequence of CiNEK6. The largest ORF frame of *CiNEK6* was found by ORF Finder server (https://www.genscript.com/sms2/orf_find.html). Multiple amino acid sequences alignment was performed by GeneDoc program and phylogenetic tree of NEK6 was created using the Neighbor-Joining algorithm from MEGA X program.

### The Plasmids Used in This Study

The ORF frame of *CiNEK6* was separately inserted into pcDNA3.1-basic, pEGFP-C1 and pCMV-Flag. The plasmids of IRF3-Flag, IRF3-GFP, IRF3-pcDNA3.1, IFN I-pro-pGL, pRL-TK were all constructed in our previous study (23). The primers used to construct recombinant plasmids of *CiNEK6* are listed in **Table 1**.

**Table 1:** Sequences and applications of the primers used in this study.

Primer name	Primer sequence (5′→3′)	Application
NEK6-F	ATGGACCAGAACAGCTTTCAAGA	cDNA cloning
NEK6-R	TTAGGTGCTGGACGTCGACA	
NEK6-3'RACE1	CCCTTTCTACAGCGACAAGAT	
NEK6-3'RACE2	CGATTACCCACCTCTGCCAT	
NEK6-5'RACE1	ACCGCGACCAATCTTTTTCT	
NEK6-5'RACE2	CCTGGTCGTTCTGTACTGGC	
Long	CTAATACGACTCACTATAGGGCAAAGCAGTGGTATCAACGCAGAGT	
Short	CTAATACGACTCACTATAGGGC	
NUP	AAGCAGTGGTATCAACGCAGAGT	
NEK6-RT-F	CGGTCAGTTCAGTGAGGTTTAT	Q-PCR
NEK6-RT-R	CATCTGGGAAAGGTCACCGG	
IFNI-RT-F	CATTGCCAACAGACGATA	
IFNI-RT-R	ATTAGCTTGCTTGATCAGATT	
β-actin-F	CACTGTGCCCATCTACGA	
β-actin-R	CCATCTCCTGCTCGAAGTC	
NEK6-pcDNA3.1-F	CGGAATTCATGGACCAGAACAGCTTTCAAGA	Eukaryotic expression vector construction
NEK6-pcDNA3.1-R	CGCTCGAGTTAGGTGCTGGACGTCGACA	
Mre11A-Flag/GFP-F	CGGAATTCAATGGACCAGAACAGCTTTCAAGA	
Mre11A-Flag/GFP-R	CGGGTACCTTAGGTGCTGGACGTCGACA	
NEK6-siRNA-183	GCAAGUGGCACUAAAGAAATT	siRNA assay
NEK6-siRNA-270	GCAACUGAACCAUCCAAAUTT	
NEK6-siRNA-446	GCAGCGCGCUGGAACACAUTT	
Negative control (N.C)	UUCUCCGAACGUGUCACGUTT	

### qRT-PCR

Grass carp were injected with 100 μl of 10^−8^ TCID_50_ GCRV or the same volume of PBS, and 24 h later, the tissues of intestines, liver, spleen and kidney from three grass carps were individually extracted. Total RNA of each tissue (50 mg) was obtained using RNA simple Total RNA Kit (Tiangen, China) and gDNA Eraser Perfect Real Time Kit (TaKaRa, Japan). The cDNA was synthesized form 1 μg of RNA using PrimeScript RT Reagent Kit (TaKaRa, Japan). The qRT-PCR reactions were as follows: 10 μl of TB Green premix Ex Taq (TaKaRa, Japan), 0.4 μl of each primer, and 7.2 μl of ddH_2_O. The cycling systems were as follows: 1 cycle of 5 min at 95°C, followed by 40 cycles of 30 s at 94°C, 30 s at 53°C, and 30 s at 72°C. The mRNA level of *CiNEK6* was detected *via* CFX Connect™ Real-Time System (Bio-Rad, USA) with TB Green Real-Time PCR Master Mix (TaKaRa, Japan). CIK cells treated with 2 μg of poly I:C or 50 μl of 10^−8^ TCID_50_ GCRV were separately cultured in 6-well plates (70% density of cells) (NEST Biotechnology, China) for 6, 12, 24, 48, and 72 h. Total RNA of the cells were harvested and *CiNEK6* mRNA level was detected as described above. NEK6 mRNA level was analyzed by comparative CT method (2^−ΔΔCT^ method). The mRNA level of *CiNEK6* is relative to *β-actin*, which is generally used as a reliable normalizer gene (24, 25). Each pair of primers used in qRT-PCR is shown in **Table 1**.

### Abs and Western Blot

Rabbit anti-IFN I and anti-GAPDH antibodies were prepared in our previous study (23). IRF3 rabbit polyclonal antibody (Cat#AF5210) and phospho-IRF3 (Ser386) rabbit monoclonal antibody (Cat#AF1594), which can be used in zebrafish according to the manufacturer’s instructions, were purchased from Beyotime (China). Mouse anti-Flag (Cat#F1804) and anti-GFP antibodies (Cat#M20004) were purchased from Sigma (USA) and Abmart (USA), respectively. Alexa Fluor**^®^**594 labeled anti-mouse IgG (Cat#ZF0513) was purchased from ZSGB-BIO (China).

The cell protein was obtained and its concentration was determined using Enhanced BCA Protein Assay Kit9 (Beyotime, China), and then the rest of protein was boiled in 5 × SDS loading buffer for 10 min. The protein was separated on 12% polyacrylamide SDS-PAGE (Bio-Rad, USA) and transferred to a nitrocellulose membrane (Millipore, USA), and then the membrane was blocked with 5% nonfat dry milk. Primary antibodies were diluted at 5% nonfat milk and incubated with membrane overnight at 4°C. Non-specific binding was washed by 1× TBST, and membrane was incubated with horseradish peroxidase conjugated secondary antibody (ZSGB-BIO, China). Finally, the membrane was imaged using a chemiluminescence imaging system (CLINX, China).

### Dual-Luciferase Reporter Assay

CIK cells in 24-well plates were used to perform dual-luciferase reporter assay. Transfection system was as follows: 0.525 μg of plasmids (0.25 μg of NEK6-pcDNA3.1, 0.25 μg of IFN I-pro-pGL and 0.025 μg of pRL-TK) and 1.58 μl of FuGENE^®^6 (Promega, USA) were mixed completely and incubated with 100 μl M199 medium for 15 min. Then, the mixture was added into the cells. At 24 h post-transfection, the cells were treated with poly I:C. Then the cells were harvested and detected using Luminoscan system (Thermo Fisher Scientific, USA).

### RNAi Assay

RNAi assay was performed in CIK cells. The siRNA against *CiNEK6* and negative control (N.C) were synthesized by GenePharma (China) (**Table 1**). CIK cells in 6-well plates were separately transfected with 2 μg of siRNA-NEK6-183, 2 μg of siRNA-NEK6-207, 2 μg of siRNA-NEK6-446, and 2 μg of N.C. 6 μl of HiperFect Transfection Reagent (QIAGEN, USA) and 2 μl of siRNA were incubated in 100 μl of M199 medium for 15 min. Then, the mixture was added into the cells. At 24 h after the transfection, the cells were stimulated with PBS or poly I:C for 12 h. The expression levels of CiNEK6 and IFN I were examined by Western blot and qRT-PCR.

### Analysis of Subcellular Localization of CiNEK6 in CIK Cells

The CIK cells were inoculated in 35-mm dishes and transfected with 2 μg of NEK6-GFP. The detailed protocol for the transfection was similar to dual-luciferase assay. At 24 h post-transfection, the cells were treated with PBS or poly I:C followed by the incubation for another 12 h. Then the cells were washed, fixed, dyed with DAPI and imaged using a confocal microscope (Leica, Germany).

### Co-Immunoprecipitation (co-IP) and Immunofluorescence Assays

CO cells were used in co-IP assay due to its high transfection efficiency. The cells were inoculated in 10-cm dishes and co-transfected with 2 μg of IRF3-GFP and 2 μg of NEK6-Flag. The detailed protocol for the transfection was similar to dual-luciferase assay. At 24 h post transfection, the cells were treated with PBS or poly I:C and subjected to incubation for another 12 h. Then the cell lysis was harvested and separately incubated with anti-Flag, anti-GFP, and IgG conjugated agarose. Anti-Flag and IgG conjugated agarose were purchased from Sigma (USA). Anti-GFP conjugated agarose was purchased from KT-HEALTH (China). According to the manufacturer’s directions, heavy chains can be detected on Flag Ab tagged agarose or IgG tagged agarose, while GFP Ab tagged agarose in unable to be detected.

In immunofluorescence assay, CIK cells were inoculated in 35-mm dishes and co-transfected with 2 μg of IRF3-Flag and 2 μg of NEK6-GFP. The detailed protocol for the transfection was similar to dual-luciferase assay. At 24 h post-transfection, the cells were treated with PBS or poly I:C and continued to be cultured for a further 12 h. The cells were subsequently washed, fixed, and incubated with anti-Flag antibody overnight. Finally, IRF3-Flag in cells were dyed with mouse Alexa Fluor**^®^**594 IgG.

### Cell Viability Assay and Crystal Violet Staining

CIK cells were inoculated in 35 mm dishes and separately transfected with 2 μg of basic-pcDNA3.1, 2 μg of IRF3-pcDNA3.1, and 2 μg of NEK6-pcDNA3.1. Twenty-four hours later, the cells in each dish were treated with 50 μl of 10^−8^ TCID_50_ for 24 h. In crystal violet staining experiment, the cells were fixed and stained with 1% crystal violet for 30 min. Cytopathic effects (CPEs) can be clearly observed. In cell viability assay, each well was added with 10 μl of CCK8 reagents (Transgene, China). Two hours later, the absorbance of each well was examined at 450 nm wave length. The detailed method of calculating cell viability was described in a previous study (26). The viral gene of GCRV, Vp5 (Genbank ID: JQ782742), was detected by qRT-PCR.

### Statistical Analysis

Each data of qRT-PCR, dual-luciferase and cell viability assays were presented as mean and ± SD (n = 3). The statistical analysis was performed using GraphPad Prism 6.0. Significant differences were analyzed by one-way analysis of variance (ANOVA) followed by Turkey’s multiple comparison tests (*p<0.05, **p<0.01). Each figure of Western blot, confocal microscopy and crystal violet staining was produced using Image J, which was represented by three independent experiments in this paper.

## Results

### Amino Acid Sequences Alignment and Homology Analysis of NEK6

**Figure 1:**
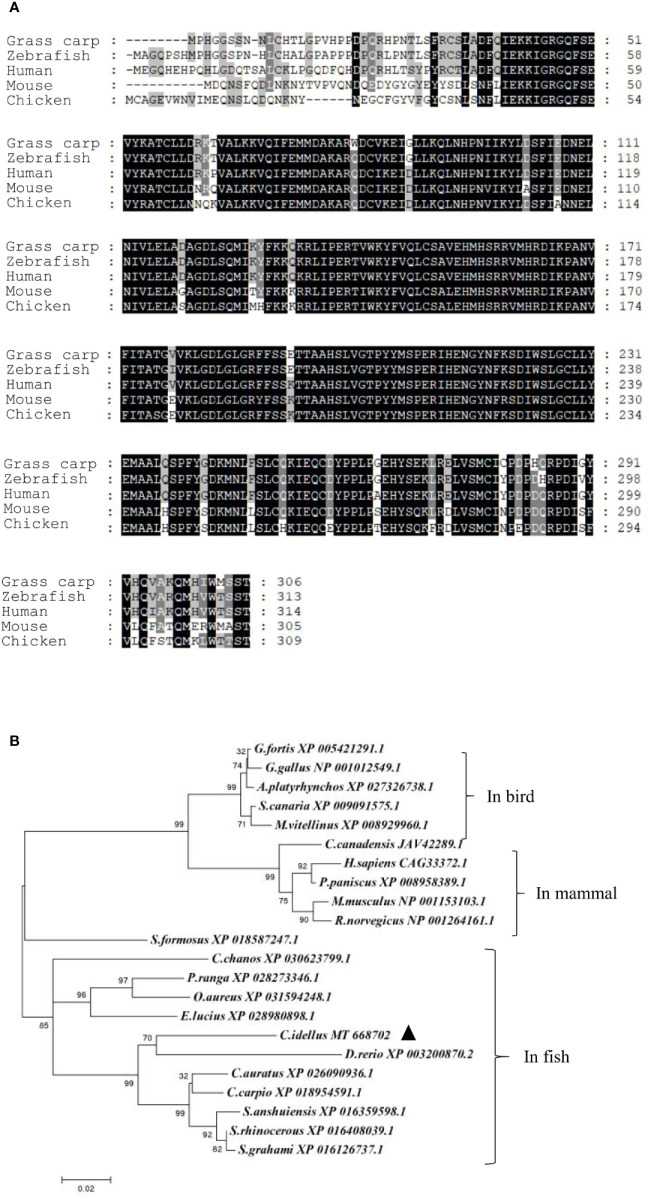
Amino acids sequences alignment and phylogenetic tree of NEK6. Amino acids sequences of grass carp NEK6, zebrafish NEK6, human NEK6 and mouse NEK6 were aligned by Gene doc server **(A)**. The phylogenetic tree of NEK6 in bird, mammals and fish was analyzed by MEGA X **(B)**.

A cDNA sequence of NEK6 was cloned in *C. idellus* (MT668702). The full-length cDNA is 1517 bp, which contains 918 bp of ORF, 241 bp of 5′ UTR and 358 bp of 3′ UTR. Multiple alignment of amino acid sequences and phylogenetic tree construction demonstrated that NEK6 is well conserved among all species studied, including fish, birds and mammals. In line with our expectation, CiNEK6 shares a high level of amino acid homology with *Danio rerio* NEK6 (**Figure 1**).

### The Expression Profiles of CiNEK6 in Tissues and CIK Cells

**Figure 2:**
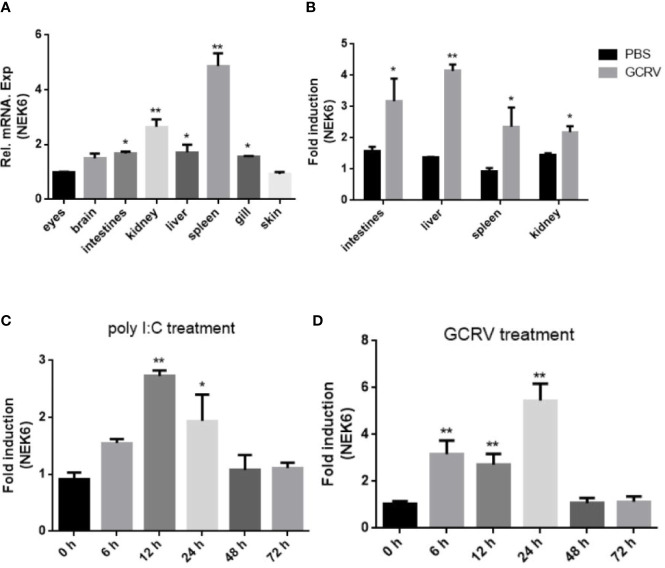
The expression of *CiNEK6* is up-regulated under GCRV and poly I:C treatment. The mRNA levels of *CiNEK6* in grass carp tissues of eyes, brain, intestines, kidney, liver, spleen, gill and skin were detected at normal condition. The expression of CiNEK6 in eyes served as a control **(A)**. The tissues of intestines, kidney, liver and spleen were chosen to detect mRNA level of *CiNEK6* at 24 h post-transfection after treatment with GCRV or PBS. The group of PBS treatment was a control **(B)**. *CiNEK6* mRNA in CIK cells was monitored at different time point (0, 6, 12, 24, 48, and 72 h) of poly I:C **(C)** and GCRV **(D)** treatment. The group of 0 h was a control. *p < 0.05, **p < 0.01.

The tissue expression profiles of CiNEK6 showed that *CiNEK6* was ubiquitously expressed, with relatively higher levels of expression in intestines, liver, spleen and kidney in comparison with those in other tissues (**Figure 2A**). Therefore, these four types of tissue were extracted from fish after treatment with GCRV or PBS. The results showed that the expression levels of *CiNEK6* were higher in GCRV-treated fish than those in PBS-treated ones (**Figure 2B**). Moreover, the mRNA level of *CiNEK6* was up-regulated in CIK cells following stimulation with poly I:C or GCRV (**Figures 2C, D**), implying that dsRNA (poly I:C and GCRV) can activate *CiNEK6* expression. In subsequent experiments, poly I:C was used as a potent stimulator of gene expression.

### CiNEK6 Suppresses IFN I Expression Upon Treatment With Poly I:C

**Figure 3:**
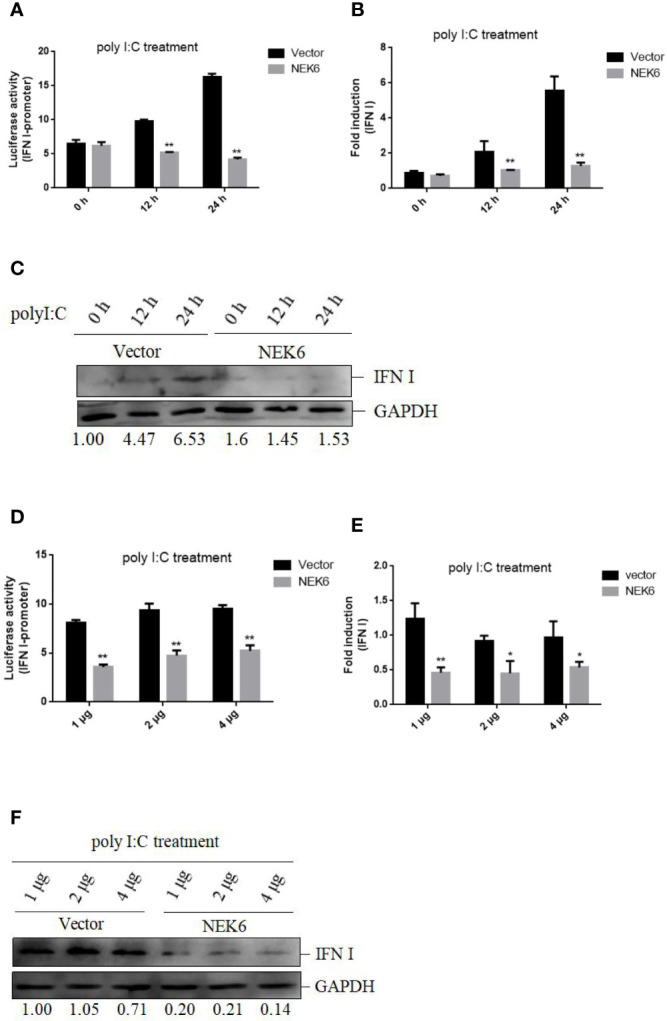
CiNEK6 suppresses IFN I expression under poly I:C treatment. CIK cells were transfected with 2 μg of basic-pcDNA3.1 (Vector) and 2 μg of NEK6-pcDNA3.1, then they were treated with poly I:C for 0, 12, and 24 h. Dual-luciferase **(A)**, qRT-PCR **(B)** and Western blot **(C)** assays were used to detect IFN I expression. CIK cells were separately transfected with basic-pcDNA3.1 (Vector) and NEK6-pcDNA3.1 at three different dosages (1, 2, or 4 μg). Then the cells treated with poly I:C were incubated for 24 h. Dual-luciferase **(D)**, qRT-PCR **(E)** and Western blot **(F)** assays were used to detect IFN I expression. The molecular masses of IFN I and GAPDH are 18 and 38 kD, respectively. Image J was used to quantify relative protein expression levels (IFN I/GAPDH). *p < 0.05, **p < 0.01.

**Figure 4:**
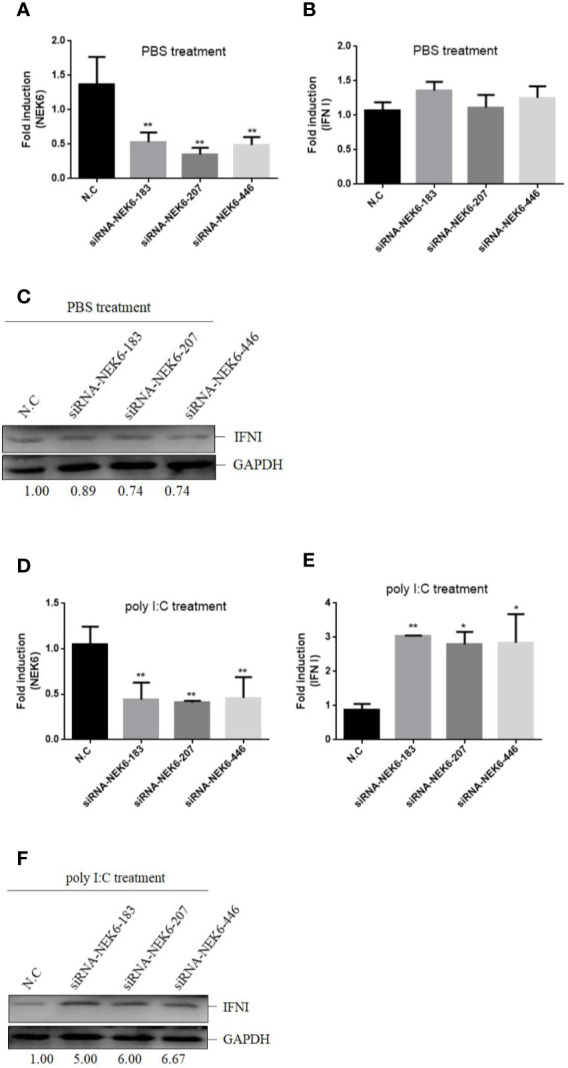
Knockdown of CiNEK6 increases IFN I expression under poly I:C treatment. CIK cells were separately transfected with 2 μg of siRNA-NEK6-183, 2 μg of siRNA-NEK6-207, 2 μg of siRNA-NEK6-446, and 2 μg of negative controls (N.C). Then the cells were treated with PBS or poly I:C. The effect of knockdown was examined through qRT-PCR **(A**, **D)**. QRT-PCR **(B**, **E)** and Western blot **(C**, **F)** were used to monitor IFN I expression. *p < 0.05, **p < 0.01.

To confirm the roles of CiNEK6 in IFN I response, IFN I expression was detected in CIK cells transfected with CiNEK6. The dual-luciferase assay, qRT-PCR and Western blot all showed that the extent of inhibition of IFN I expression was gradually increased following treatment with increased dosage and prolonged duration of poly I:C (**Figure 3**). In contrast, knockdown of CiNEK6 increased IFN I expression in poly I:C-treated cells, but not in PBS-treated cells (**Figure 4**). These data suggested that CiNEK6 plays an antagonistic role in IFN I response under treatment with poly I:C.

### CiNEK6 Is Translocated From the Cytoplasm to the Periphery of the Nuclear Membrane Under Treatment With Poly I:C or GCRV

**Figure 5:**
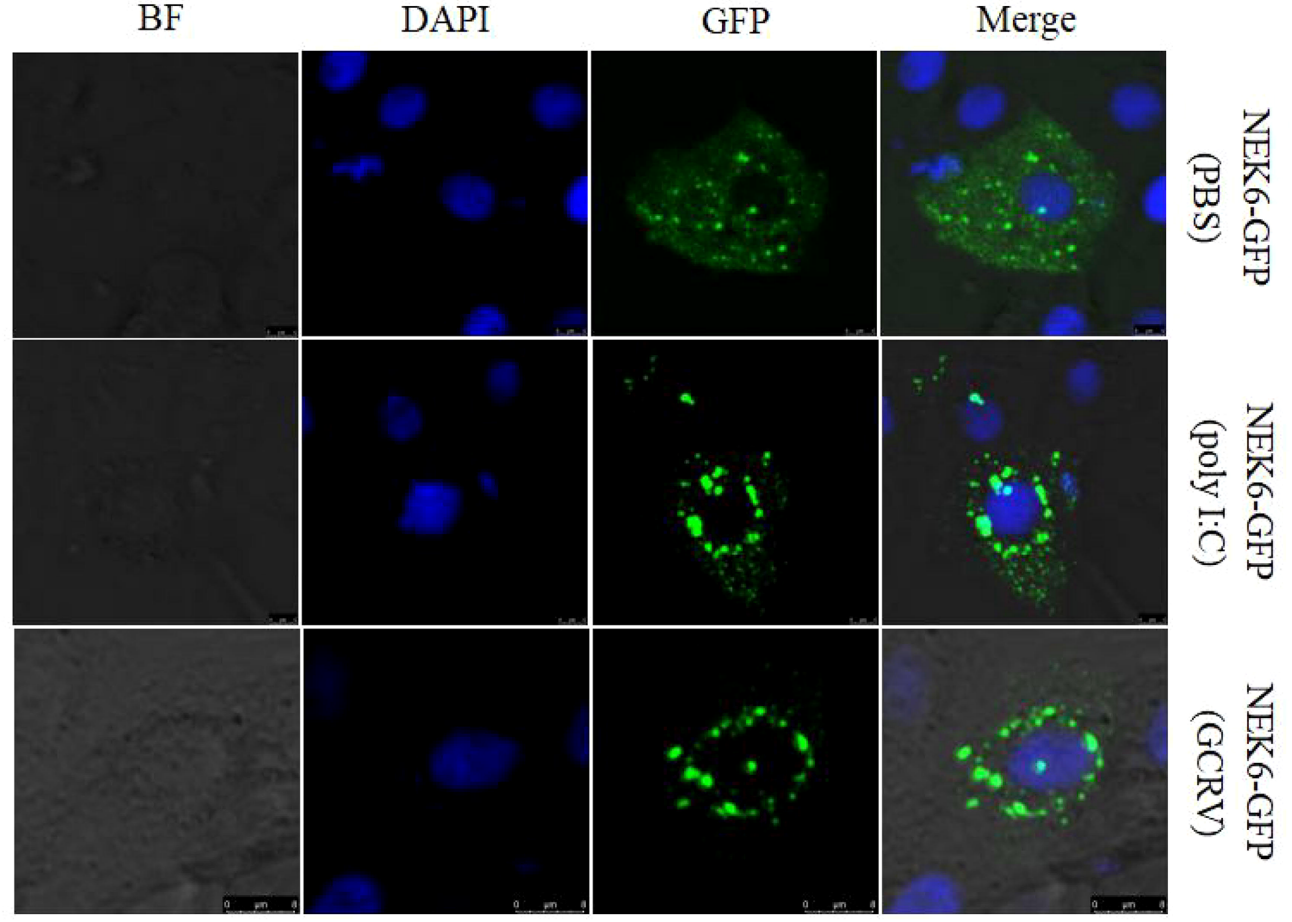
Subcellular localization of CiNEK6 in CIK cells. After CIK cells were transfected with 2 μg of NEK6-GFP plasmids, and the cells were treated with PBS or poly I:C and incubated for another 12 h. Then the cells were washed, fixed, and dyed. The localization of CiNEK6 in CIK cells were captured by a confocal microscope. The scale bar is 5 µm. Magnification of cells is × 400.

It is known that protein location within the cell may define its function. The CIK cells were transfected with NEK6-GFP, and then the transfected cells were divided into three groups, which were separately treated with PBS, poly I:C and GCRV. CiNEK6-GFP protein was evenly distributed in the cytoplasm in PBS-treated groups; however, CiNEK6-GFP protein was mainly congregated at the periphery of nuclear membrane in poly I:C or GCRV-treated group (**Figure 5**). The data further suggested that CiNEK6 can respond to the stimulation with poly I:C or GCRV.

### CiNEK6 Interacts With CiIRF3 After Treatment With Poly I:C

**Figure 6:**
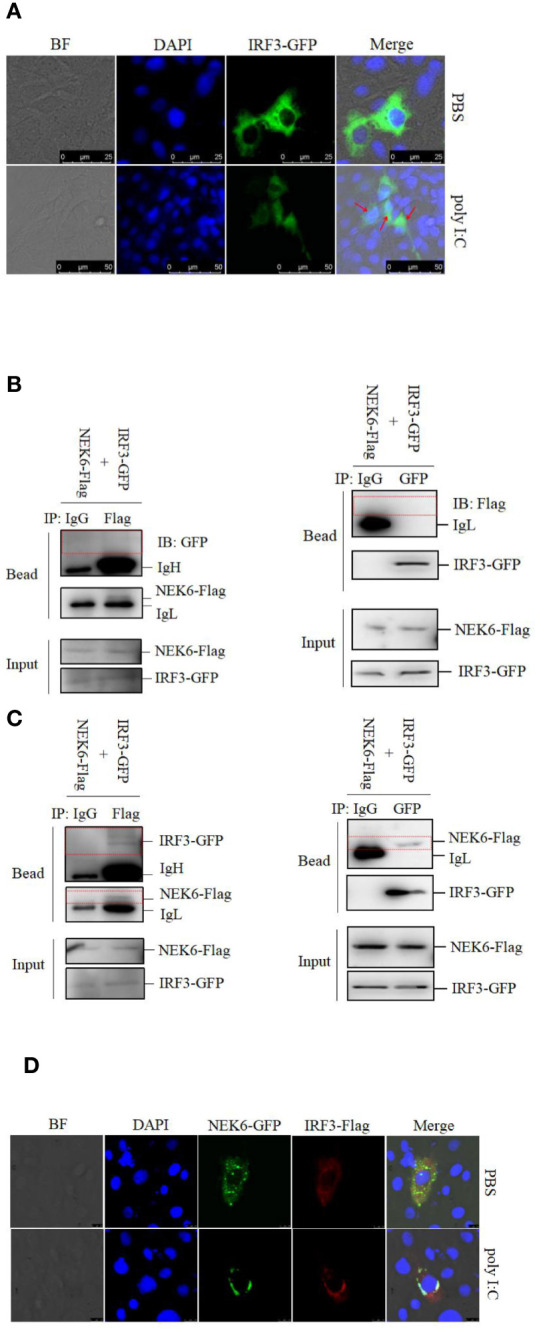
CiNEK6 interacts with CiIRF3 in the cytoplasm. In subcellular localization of IRF3, CIK cells were transfected with 2 μg of IRF3-GFP. Then, the cells were treated with PBS or poly I:C. The subcellular localization of IRF3 in CIK cells was imaged by a confocal microscope **(A)**. The scale bar is 25 µm. Magnification of cells is × 400. In co-IP assay, the plasmids of NEK6-Flag and IRF3-GFP were co-transfected into CO cells. At 24 h post-transfection, the cells were treated with PBS **(B)** or poly I:C **(C)**. Each group was divided into two panels. Left panel of immunoprecipitation was conducted by anti-GFP antibody and right panel was anti-Flag antibody. The immunoprecipitation of IgG was selected as a negative control. The molecular weight of NEK6-Flag, IRF3-GFP, IgH and IgL are 38, 85, 58, and 35 kD, respectively. In co-localization assay, 2 μg of NEK6-GFP and 2 μg of IRF3-Flag were co-transfected into CIK cells. At 24 h post-transfection, the cells were treated with PBS or poly I:C. The co-localization of NEK6 and IRF3 was imaged by a confocal microscope **(D)**. The scale bar is 75 µm. Magnification of cells is ×400.

It is clear that IRF3 is apt to translocate from the cytoplasm into the nucleus upon treatment with poly I:C (**Figure 6A**). To further explore the NEK6-mediated pathway, co-IP assay was performed in CO cells. CiNEK6 failed to interact with CiIRF3 in PBS-treated cells (**Figure 6B**); however, the interaction of CiNEK6 and CiIRF3 was detected in poly I:C-treated cells (**Figure 6C**).

To determine the subcellular localization of CiNEK6-CiIRF3 complex, CIK cells were co-transfected with NEK6-GFP and IRF3-Flag. In poly I:C-treated cells, NEK6-GFP was co-localized with IRF3-Flag at the periphery of the nuclear membrane (**Figure 6D**). The results suggested that CiNEK6 may suppress the cytoplasm-to-nucleus translocation of IRF3 under treatment with poly I:C.

### CiNEK6 Inhibits IRF3-Induced IFN I Expression

**Figure 7:**
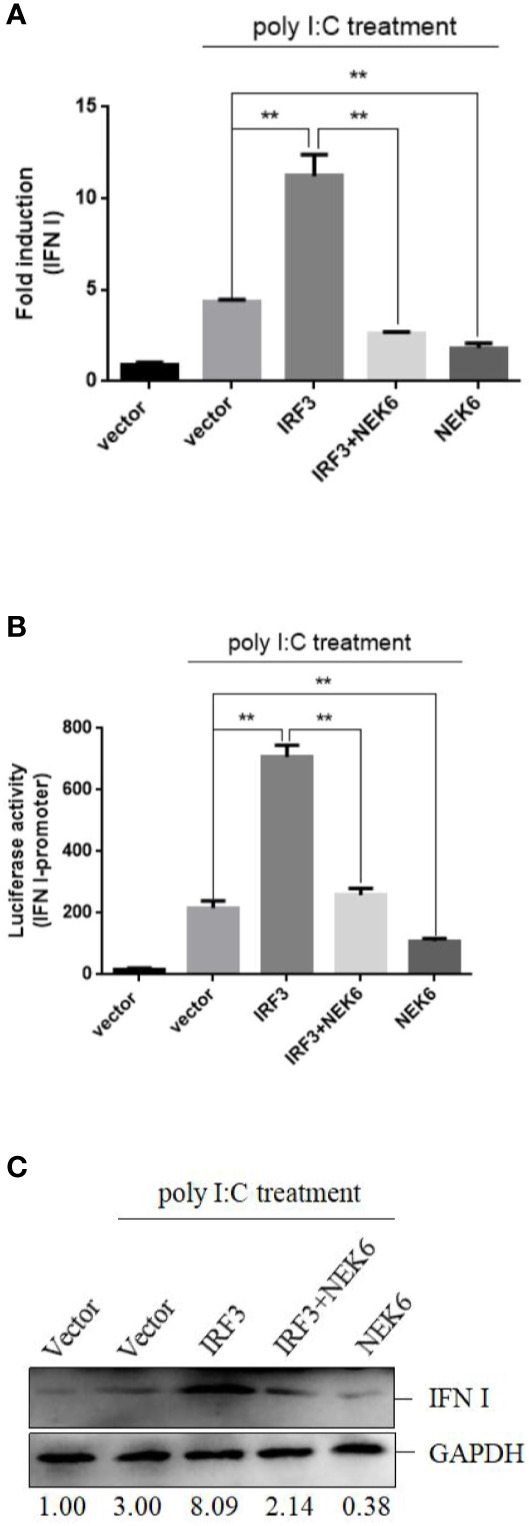
CiNEK6 blocks CiIRF3-induced IFN I expression. CIK cells were single transfected or co-transfected with 2 μg of NEK6-pcDNA3.1 and 2 μg of IRF3-pcDNA3.1. The cells transfected with 2 μg of basic-pcDNA3.1 were performed as negative controls. At 24 h post-transfection, the cells were treated with poly I:C. Dual-luciferase assay **(A)**, qRT-PCR **(B)** and Western blot **(C)** were used to examine IFN I expression. **p < 0.01.

**Figure 8:**
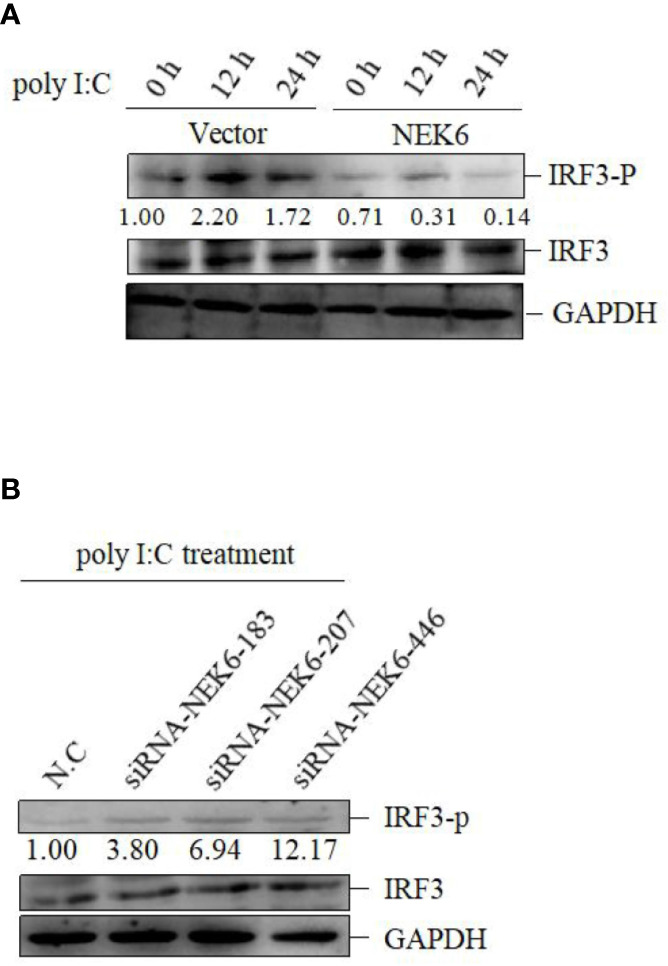
CiNEK6 inhibits the phosphorylation of IRF3 under poly I:C treatment. CIK cells were transfected with 2 μg of basic-pcDNA3.1 or 2 μg of NEK6-pcDNA3.1. At 24 h post-transfection, the cells were treated with poly I:C and incubated for 0, 12, and 24 h **(A)**. CIK cells were transfected with 2 μg of siRNA-NEK6-183, 2 μg of siRNA-NEK6-207 or 2 μg of siRNA-NEK6-446 and 2 μg of N.C. At 24 h post-transfection, the cells were treated with poly I:C **(B)**. Phosphorylation of IRF3 (IRF3-p) was detected.

In subsequent experiment, the interplay between CiNEK6 and CiIRF3 was investigated. CIK cells were transfected with pcDNA3.1-NEK6 or co-transfected with both of pcDNA3.1-NEK6 and pcDNA3.1-IRF3. Transfection of basci-pcDNA3.1 was used as a negative control. The results of dual-luciferase assay, qRT-PCR and Western blot suggested that CiIRF3 activated the IFN I expression, but CiNEK6 blocked CiIRF3-induced IFN I expression (**Figure 7**). CiNEK6 inhibited IRF3 phosphorylation in CIK cells upon stimulation with poly I:C (**Figure 8A**). In contrast, knockdown of CiNEK6 promoted IRF3 phosphorylation in CIK cells (**Figure 8B**). These results indicated that CiNEK6 inhibits IFN I expression through suppressing IRF3 phosphorylation.

### CiNEK6 Enhances GCRV Proliferation in CIK Cells

**Figure 9:**
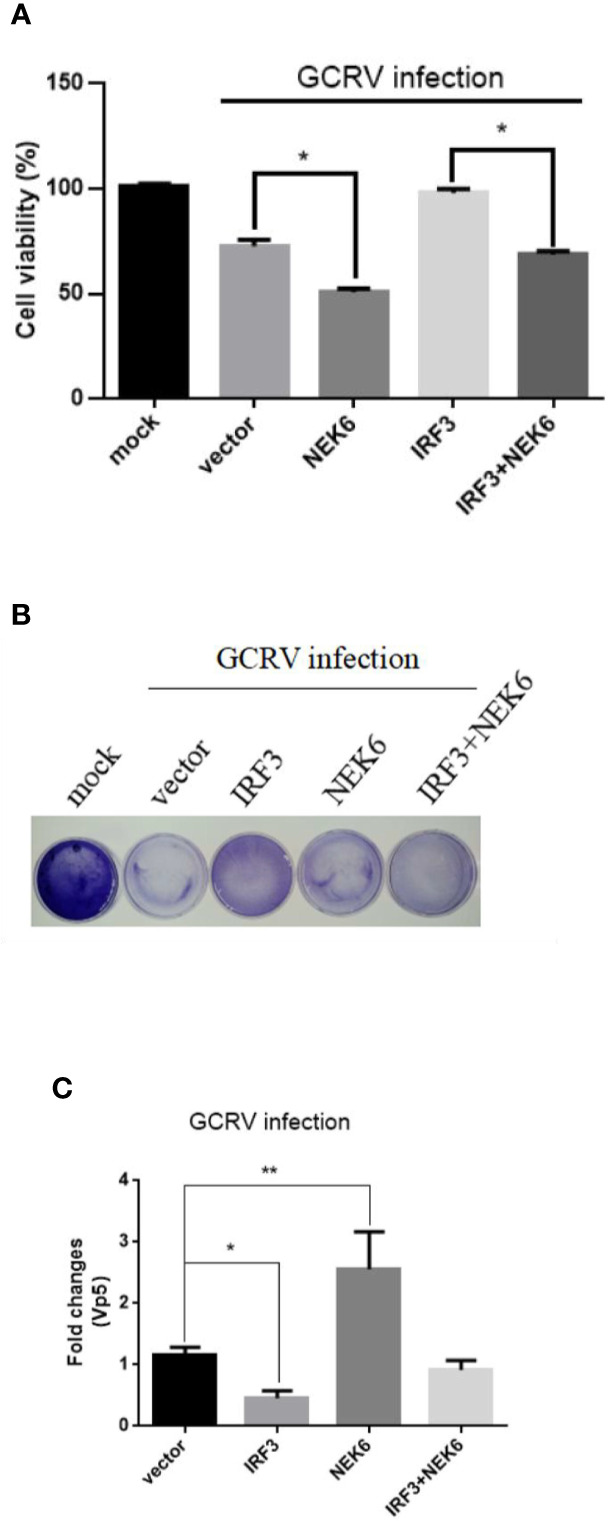
CiNEK6 enhances GCRV proliferation in CIK cells. CIK cells were separately transfected with 2 μg of NEK6-pcDNA3.1 and 2 μg of IRF3-pcDNA3.1. At 24 h post-transfection, the cells were treated with GCRV and continue to be cultured for 24 h. This experiment was divided into three groups. One group of cell viability in 96-well plates was analyzed by CCK assay **(A)**. The second group of cells in 35-cm^2^ dishes was stained with crystal violet for CPEs **(B)**. The third group of cells in six-well plates was used to abstracted total RNA, the mRNA level of Vp5 was detected by qRT-PCR **(C)**. *p< 0.05, ** p < 0.01.

Although CiNEK6 inhibited IRF3-induced IFN I response, it is necessary to explore how CiNEK6 responds to GCRV challenge. Cell viability and crystal violet staining assays were performed in CIK cells. Overexpression of CiNEK6 notably reduced cell viability and impeded the protective effect of IRF3 on the cells under infection with GCRV (**Figure 9A**). Crystal violet staining and detection of Vp5 also showed that CiNEK6 enhanced the proliferation of GCRV in CIK cells (**Figures 9B, C**).

## Discussion

NEK6 has been considered to be involved in cell growth, apoptosis, transformation and tumorigenesis (27, 28). Some previous researches have also shown that NEK6 participates in many cellular signaling pathways. However, the detailed mechanism by which NEK6 modulates immune response is still unknown in vertebrates. In this study, we preliminarily revealed that CiNEK6 is a negative regulator of dsRNA-induced IFN I response.

In vertebrates, the amino acid sequences of NEK6 proteins are very much alike (**Figure 1**), suggesting that the function of NEK6 may be relatively highly conserved. CiNEK6 was ubiquitously expressed in all tested tissues, with higher levels of expression detected in intestines, liver, spleen and kidney than that in other tissues under treatment with GCRV (**Figures 2A, B**), demonstrating that CiNEK6 is engaged in GCRV-induced cellular signaling pathway. The same conclusion was also obtained in CIK cells (**Figures 2C, D**). Therefore, CiNEK6 is presumed to be involved in innate immune response. Unexpectedly, CiNEK6 inhibits IFN I expression only under treatment with poly I:C (**Figures 3**, **4**). Generally, IFN I response remains inactivated under normal conditions, and NEK6 activity is not required. These results suggest that CiNEK6 may act as an immunomodulator to regulate excessive expression of IFN I in innate immune response. The enhanced expression of CiNEK6 upon stimulation with poly I:C results in the down-regulation of innate immune responses. Similarly, the expression levels of zebrafish FOXO3 and grass carp IRF2 are elevated after stimulation with SVCV and poly I:C, respectively; and then zebrafish FOXO3 and grass carp IRF2 inhibit IRF3/IRF7-mediated and IRF1-mediated immune response, respectively (19, 29).

Under normal circumstances, CiNEK6 protein is dispersed in the cytoplasm; however, they are congregated at the periphery of the nuclear membrane after treatment with poly I:C or GCRV (**Figure 5**). The migration of CiNEK6 may be essential for its activity. Similarly, MITA is translocated from the ER or the mitochondria to the Golgi apparatus upon sensing cytosolic viral DNA (30–33). Grass carp DDX41 and SAMHD1 are located in the nucleus, but they are translocated from the nucleus to the cytoplasm in response to virus infection (34, 35). Of course, the mechanism of the nucleocytoplasmic transport of CiNEK6 needs to be further studied. We suspect that CiNEK6 tends to congregate at the periphery of the nuclear membrane to prevent the cytoplasm-to-nucleus translocation of the transcription factors.

It is acceptable that the nuclear-translocation and phosphorylation are necessary for the activation of IRF3 (36, 37). The inactivated CiIRF3 was located in the cytoplasm; however, the activated CiIRF3 was translocated from the cytoplasm to the nucleus in response to poly I:C treatment (**Figure 6A**). CiNEK6 interacted with CiIRF3 only under treatment with poly I:C (**Figures 6B, C**), and then the binding suppressed the cytoplasmic-to-nuclear translocation of IRF3 (**Figure 6D**). These results further confirm our previous speculation about the reason that underlines the translocation of CiNEK6.

CiNEK6 inhibited IFN I expression *via* controlling IRF3 (**Figures 7A–C**). Moreover, CiNEK6 inhibited the phosphorylation of IRF3 upon treatment with poly I:C (**Figures 8A, B**). However, the mechanism needs to be elucidated in our future study. DDX56 is a negative regulator for virus-induced type I IFN expression, which suppresses the nuclear translocation and phosphorylation of IRF3 (38). Lysine acetyltransferase 8 (KAT8) directly interacts with IRF3 and promotes IRF3 acetylation and then inhibits its activity (39). CiNEK6 also inhibited the antiviral response (**Figures 9A–C**). Generally, CiNEK6 suppresses innate immune response in a negative feedback loop, which maintains the cell homeostasis.

Taken together, the data revealed that CiNEK6 blocked IFN I expression through inhibiting the activity of IRF3 under treatment with poly I:C. More investigation about the inhibitory mechanism of CiNEK6 will further elucidate the immunoregulatory signaling pathway in cells.

## Data Availability Statement

The datasets presented in this study can be found in online repositories. The names of the repository/repositories and accession number(s) can be found below: https://www.ncbi.nlm.nih.gov/genbank/, MT668702.

## Ethics Statement

The animal study was reviewed and approved by Nanchang University.

## Author Contributions

CH and ZD supervised the research. XX conceived the study, designed, and performed the experiments. ML, XX, and JH analyzed the experiments and data. ZJ, KC, and YL provided reagents, technical assistance, and contributed to completion of the study. XX wrote the manuscript. All authors contributed to the article and approved the submitted version.

## Funding

The study was supported by the earmarked fund for the Project Funded by China Postdoctoral Science Foundation (2019M662279), Research Project Funded by Jiangxi Postdoctoral Science Foundation (2019KY43) and Jiangxi Agriculture Research System (JXARS-04).

## Conflict of Interest

The authors declare that the research was conducted in the absence of any commercial or financial relationships that could be construed as a potential conflict of interest.
