# Supplementary material for: Grass Carp (Ctenopharyngodon idellus) NIMA-Related Kinase 6 Blocks dsRNA-Induced IFN I Response by Targeting IRF3
Source: Front Immunol. 2021 Jan 8;11:597775. doi: 10.3389/fimmu.2020.597775 (PMC7820699; doi:10.3389/fimmu.2020.597775)
Supplement: Supplementary file 1 [file DataSheet_1.doc]

Supplementary Figure 1


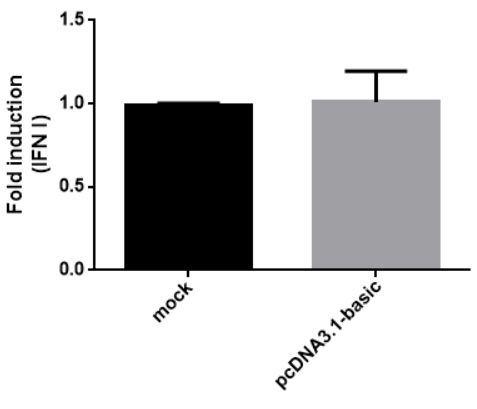


CIK cells were seeded in 6-well plates and transfected with 2 μg of pcDNA3.1-basic, and the mock was treated with the same volume of transfection reagent. After 24 h, qRT-PCR was used to detect IFN I expression.
